# Rare Bilateral Symmetrical Kimura Disease: Diagnostic Challenges and Treatment Success in a 63-Year-Old Male Patient

**DOI:** 10.7759/cureus.101191

**Published:** 2026-01-09

**Authors:** Yuhui Liu, Yong Huang

**Affiliations:** 1 Department of Radiology, Shandong Cancer Hospital and Institute, Shandong First Medical University and Shandong Academy of Medical Sciences, Jinan, CHN

**Keywords:** cervical lymphadenopathy, eosinophilia, kimura disease, magnetic resonance imaging, retroauricular mass

## Abstract

Kimura disease (KD) is a rare chronic inflammatory disorder that presents with eosinophilic infiltration and angiolymphoid proliferation, often affecting young men. However, its clinical manifestations can be elusive, especially when presenting bilaterally, which is exceptionally rare. This case report details a 63-year-old male patient with a 30-year history of progressive bilateral retroauricular masses, pruritus, and skin hyperpigmentation, a presentation that posed significant diagnostic challenges. Magnetic resonance imaging (MRI) revealed poorly defined, heterogeneously enhancing masses and bilateral cervical lymphadenopathy. The diagnosis was confirmed through histopathology and peripheral eosinophilia. The patient was successfully treated with surgical excision and corticosteroid therapy. This case underscores the importance of considering KD in the differential diagnosis of chronic, bilateral neck masses, particularly when accompanied by eosinophilia, and highlights the role of imaging and histopathology in accurate diagnosis.

## Introduction

Kimura disease (KD) is a rare chronic inflammatory disorder characterized by painless subcutaneous masses, tissue eosinophilia, and frequently elevated serum IgE levels, most commonly affecting young Asian men and predominantly involving the head and neck region [1]. Bilateral or symmetrical involvement is exceptionally uncommon and may closely resemble lymphoproliferative or malignant disease, resulting in diagnostic delay or misdiagnosis. Imaging modalities such as magnetic resonance imaging (MRI) and computed tomography (CT) play a crucial role in characterizing lesion morphology, assessing regional lymphadenopathy, and narrowing the differential diagnosis in conjunction with clinical and laboratory findings [2].

Here, we report a rare case of bilateral retroauricular KD in a 63-year-old man with a 30-year disease history. We highlight the characteristic imaging features and clinicopathologic correlation and discuss key points for differentiating KD from malignancy in patients with long-standing bilateral neck masses.

## Case presentation

Clinical history

A 63-year-old male farmer presented to our institution with progressively enlarging bilateral neck masses localized to the retroauricular regions. The patient reported that small, painless nodules first appeared behind both ears approximately 30 years earlier, initially the size of soybeans. Over the subsequent decades, the nodules gradually enlarged to soft, egg-sized masses. During the year prior to presentation, he noted worsening pruritus over the lesions and progressive cutaneous hyperpigmentation, leading to significant cosmetic concern.

He denied pain, constitutional “B symptoms” (fever, night sweats, and weight loss), salivary gland dysfunction, or systemic allergic symptoms. The patient had no history of autoimmune disease, malignancy, or parasitic infection. He had been repeatedly diagnosed elsewhere with “benign lymphadenopathy” and received only intermittent symptomatic treatment, without definitive evaluation or tissue diagnosis.

Physical examination

**Figure 1:**
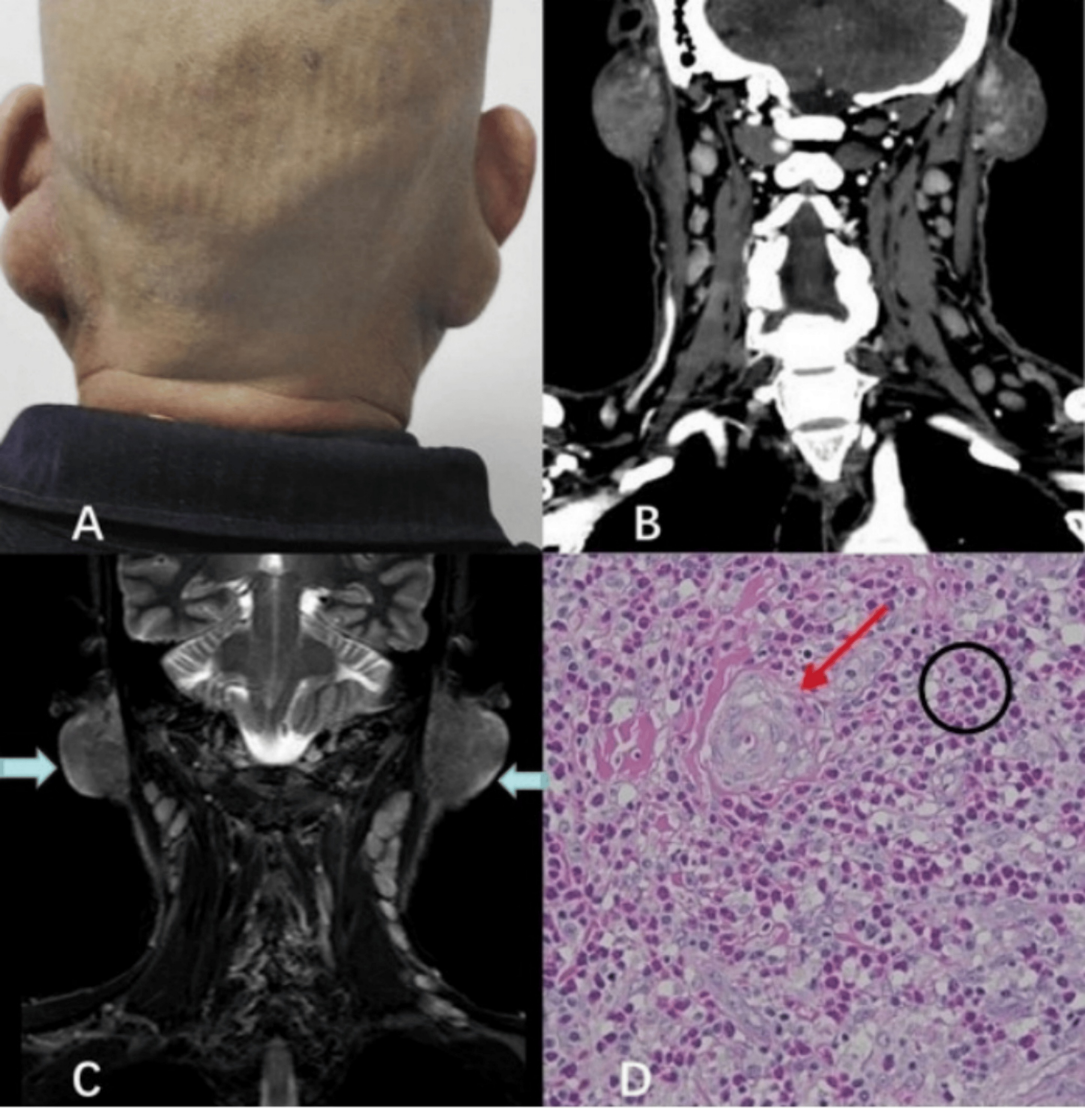
Clinical, radiological, and histopathological features of the patient (A) Clinical photograph showing bilateral retroauricular soft-tissue masses with overlying lichenified, hyperpigmented skin. (B) Coronal contrast-enhanced CT of the neck confirming bilateral retroauricular soft-tissue masses and cervical lymphadenopathy. (C) Coronal STIR sequence demonstrating bilateral retroauricular lesions with heterogeneous "cotton-wool"-like hyperintensity (blue arrows), with poor demarcation from the adjacent parotid glands. (D) Histopathologic image consistent with Kimura disease. The red arrow indicates the proliferation of postcapillary venules lined by plump endothelial cells, and the black circle highlights the dense eosinophilic infiltration within the interfollicular regions (hematoxylin and eosin stain). CT: computed tomography; STIR: short tau inversion recovery

On physical examination, two soft, ill-defined, non-tender masses were palpated in the bilateral posterior auricular regions. The left mass measured approximately 3.0 × 2.8 cm and the right mass 3.5 × 3.2 cm (Figure 1A). The overlying skin showed lichenification and diffuse hyperpigmentation but no ulceration, erythema, or warmth. There was no cranial nerve deficit, no palpable parotid enlargement, and no obvious systemic lymphadenopathy. Cardiopulmonary and abdominal examinations were unremarkable.

Imaging findings

Contrast-enhanced CT of the neck confirmed the presence of bilateral retroauricular soft-tissue masses with heterogeneous density and mild to moderate enhancement, along with bilateral cervical lymphadenopathy (Figure 1B). Contrast-enhanced MRI of the head and neck was performed to further characterize the lesions. The MRI demonstrated bilateral subcutaneous retroauricular lesions exhibiting heterogeneous signal intensity, characterized by T2-weighted hyperintensity and intermediate-to-low T1 signal, consistent with recent radiological descriptions of KD [3]. The lesions displayed ill-defined margins with poor tissue plane demarcation from the adjacent parotid glands, yet there was no evidence of bone erosion or aggressive invasion (Figure 1C). Additionally, multiple enlarged bilateral cervical lymph nodes were noted, with the largest node measuring approximately 1.8 cm in short-axis diameter. The long-standing bilateral retroauricular masses, characteristic MRI pattern, and associated lymphadenopathy raised suspicion of KD, though malignant lymphoma and other neoplastic processes remained major differential diagnoses.

Laboratory analysis

Routine blood tests revealed significant abnormalities consistent with the inflammatory nature of the disease. The most notable finding was marked eosinophilia, with an absolute eosinophil count of 5.29 × 10⁹/L and eosinophils accounting for 38.0% of total leukocytes. Renal function and urinalysis were normal, with no evidence of proteinuria. Although serum IgE testing was not available at the time of the initial workup, the pronounced peripheral eosinophilia strongly supported an underlying eosinophil-rich inflammatory process consistent with KD [1,3]. Detailed hematologic parameters are summarized in Table 1.

**Table 1:** Laboratory test results on admission *Values outside the reference range.

Parameter	Full name	Result	Unit	Reference range
WBC	White blood cell count	9.3	×10⁹/L	4.1-11.0
Neu#	Neutrophil count	5.84	×10⁹/L	1.8-8.3
Neu%	Neutrophil percentage	62.8	%	37-77
Lym#	Lymphocyte count	2.71	×10⁹/L	1.2-3.8
Lym%	Lymphocyte percentage	29.1	%	17-54
Mon#	Monocyte count	0.66	×10⁹/L	0.14-0.74
Mon%	Monocyte percentage	7.1	%	2-11
Eos#*	Eosinophil count	5.29	×10⁹/L	0.00-0.68
Eos%*	Eosinophil percentage	38	%	0.0-9.0
Bas#	Basophil count	0.02	×10⁹/L	0-0.07
Bas%	Basophil percentage	0.2	%	0-1
RBC	Red blood cell count	5.17	×10¹²/L	4.5-5.9
Hb	Hemoglobin	150	g/L	129-172
HCT	Hematocrit	45.1	%	39-51
MCV	Mean corpuscular volume	87.2	fL	80-100
MCH	Mean corpuscular hemoglobin	29	pg	25-34
MCHC	Mean corpuscular hemoglobin conc.	333	g/L	310-355
RDW-CV	RBC distribution width-CV	12.3	%	12.2-14.6
RDW-SD*	RBC distribution width-SD	39.3	fL	39.9-52.2
PLT	Platelet count	305	×10⁹/L	150-407
MPV	Mean platelet volume	9.9	fL	9.1-12
PDW	Platelet distribution width	11.4	fL	9.8-15.2
PCT	Plateletcrit	3	mL/L	1.9-3.6
P-LCR	Platelet large cell ratio	24.6	%	19.5-41.9

Surgical management and histopathologic findings

Given the progressive enlargement, cosmetic impact, and the need to exclude malignancy, the patient underwent bilateral surgical excision of the retroauricular masses under general anesthesia. The lesions were found to be poorly circumscribed, lobulated soft-tissue masses within the subcutaneous tissue, loosely adherent to but not infiltrating the adjacent parotid tissue. Enlarged cervical lymph nodes were also sampled. Histopathologic examination revealed prominent reactive lymphoid follicles with well-formed germinal centers and a diffuse, dense eosinophilic infiltration in the interfollicular regions (Figure 1D). Furthermore, there was a proliferation of small-caliber postcapillary venules lined by plump endothelial cells. Importantly, no cytologic atypia or features suggestive of lymphoma or metastatic malignancy were observed. These findings were consistent with KD and helped differentiate it from angiolymphoid hyperplasia with eosinophilia (ALHE), which typically shows more prominent vascular proliferation with hobnail endothelial cells and less marked lymphoid follicular hyperplasia [1,3].

Postoperative course and follow-up

The postoperative course was uneventful. Based on the confirmed diagnosis of KD and the presence of significant peripheral eosinophilia, a short course of systemic corticosteroids (oral prednisone, tapered over several weeks) was initiated to address potential subclinical disease and reduce the risk of early recurrence. At subsequent follow-up visits, the patient reported resolution of pruritus and improvement in skin hyperpigmentation. No new masses or lymphadenopathy were detected on clinical examination. Imaging follow-up is ongoing to monitor for local or regional recurrence.

## Discussion

KD is a rare inflammatory condition typically affecting young Asian men, presenting with subcutaneous masses, eosinophilia, and elevated serum IgE levels. Bilateral involvement, as seen in this case, is uncommon and can mimic malignancy or lymphoproliferative disorders, complicating diagnosis [4].

This patient’s 30-year disease course and bilateral retroauricular masses highlight the diagnostic challenges of KD, especially in older patients [4]. In contrast, pediatric cases often exhibit distinct clinical and pathological features, underscoring the broad age spectrum of KD [5]. Imaging, particularly MRI, plays a pivotal role in diagnosis; a recent retrospective analysis highlighted that KD lesions typically manifest as ill-defined subcutaneous masses with heterogeneous enhancement and T2 hyperintensity, often accompanied by adjacent subcutaneous fat atrophy [3]. The combination of elevated peripheral eosinophilia and histopathology confirmed the diagnosis of KD, differentiating it from conditions like lymphoma and ALHE [3,4].

Surgical excision is the primary treatment for KD, though recurrence is common. Corticosteroids are often used to manage inflammation and reduce recurrence risk [4]. However, for refractory or recurrent cases, recent literature suggests the efficacy of biological agents. Dupilumab (targeting IL-4/IL-13) and mepolizumab (targeting IL-5) have shown promising results in reducing mass size and eosinophilia in patients who respond poorly to conventional therapies [6]. In our patient, surgery combined with corticosteroids resulted in good short-term control, but long-term follow-up is essential for detecting potential recurrence.

## Conclusions

In conclusion, this case underscores the importance of considering KD in the differential diagnosis of chronic, bilateral neck masses, especially when accompanied by peripheral eosinophilia. A multidisciplinary approach involving clinical evaluation, imaging, histopathology, and follow-up is crucial to ensure accurate diagnosis and effective management of this rare condition.
